# Plasmonics for Telecommunications Applications

**DOI:** 10.3390/s20092488

**Published:** 2020-04-28

**Authors:** William O. F. Carvalho, J. Ricardo Mejía-Salazar

**Affiliations:** National Institute of Telecommunications (Inatel), 37540000 Santa Rita do Sapucaí, MG, Brazil; william.carvalho@dtel.inatel.br

**Keywords:** fiber interconnectors, filters, modulators, nanoantennas, nanophotonic, optical computing, photodetectors, plasmonics, routers, switches, telecommunications

## Abstract

Plasmonic materials, when properly illuminated with visible or near-infrared wavelengths, exhibit unique and interesting features that can be exploited for tailoring and tuning the light radiation and propagation properties at nanoscale dimensions. A variety of plasmonic heterostructures have been demonstrated for optical-signal filtering, transmission, detection, transportation, and modulation. In this review, state-of-the-art plasmonic structures used for telecommunications applications are summarized. In doing so, we discuss their distinctive roles on multiple approaches including beam steering, guiding, filtering, modulation, switching, and detection, which are all of prime importance for the development of the sixth generation (6G) cellular networks.

## 1. Introduction

Recent years have witnessed considerable progress in the exploitation of the THz spectrum, from the infrared to the visible, to cope with the ever-growing demand for higher-data-rates, broader bandwidths, low-power-consumption, and higher on-chip integrability for telecommunications applications [1,2,3,4,5,6,7]. In this context, the unique ability of metallic nanostructures to capture and concentrate light at subwavelength dimensions has emerged as a promising solution. In contrast to their photonic (limited in size by diffraction laws) and nanoelectronic (interconnection delays) counterparts, where signals are carried by photons or electrons, respectively, plasmonic modes are just the resonant coupling of electromagnetic waves to collective free-electron oscillations at metal surfaces. Therefore, devices with the incredible data rates of optical signals in photonic components and the extremely small sizes of electronic circuits can be developed through the synergistic integration of photonic, plasmonic, and electronic components on the same chip [8,9,10]. According to the geometrical properties of the metallic nanostructures, plasmonic resonances can involve guided surface plasmon resonances (SPRs) or localized surface plasmon resonances (LSPRs), as it will be discussed later on. The excitation and radiation properties of LSPRs can be used in wireless optical links in, for example, the optoelectronic conversion of optical-to-THz and THz-to-optical signals for the seamless integration of fibers-to-THz communication front-ends [4]. Ultrafast board-to-board and chip-to-chip communications approaches, on the other hand, are also being investigated with nanometric-length scale analogies of radio frequency (RF) antennas to work in the optical frequency range [11,12,13,14,15]. In addition to these optical-wireless nanolinks, SPRs properties can also be used for plasmonic waveguides [16,17,18,19,20], modulators [21,22], filters [23,24], switches, and routers [25,26,27].

In this review paper, we advocate the importance of plasmonic systems for ultrafast broadband telecommunications applications. We highlight the importance of plasmonic nanolinks, owing to their ability to convert free radiation into localized energy and vice versa, for the future 6G technology, where complementary advantages of optical transmission systems—virtually unlimited and reliable capacity—are of great importance [4,15]. Another important advantage of this technology, in contrast to their photonic counterparts, is the ability for high-speed optical signal transmission with device sizes below the diffraction limit, which will certainly allow for higher integration levels. The challenges associated to ohmic losses of metals at optical frequencies, limiting propagation of guided SPRs to distances of only few micrometers, are also discussed. This review starts discussing the physics behind excitation of different plasmonic resonances. Applications in nanoantennas, modulators, filters, switches, routers, optical computing, and detectors are then discussed. Finally, we conclude by summarizing and outlooking new trends in plasmonics for telecommunications applications.

## 2. Plasmonic Resonances: Fundamentals

**Figure 1:**
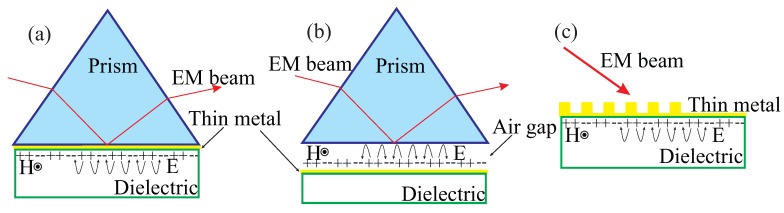
Main techniques to excite surface plasmon polaritons (SPPs): (**a**) The Kretschmann Configuration; (**b**) Otto Configuration, and (**c**) Grating Coupling.

SPRs, the resonant coupling of the electric field component of light with free-electron excitations in metal surfaces, have the ability to enhance and squeeze light fields beyond the diffraction limit [28]. These resonances can involve propagating (guided) or localized (radiating) SPRs (LSPRs), depending on the geometrical properties of the metallic surface [29,30], thus providing a path for tailoring different nanoscale near-field optical phenomena [31,32,33]. Differences are not only observed in the propagation and radiation properties, but also in the way they are excited. Whereas LSPRs can be directly excited by freely-propagating light, SPRs need a proper matching mechanism to couple free-to-guided light modes, as schematized in Figure 1. The SPR wavevector for a thick metallic film, whose thickness is larger than the SPR decaying length, is written as $k_{\mathrm{SPR}}=k_{0}\sqrt{\frac{\varepsilon_{m}\varepsilon_{d}}{\varepsilon_{m}+\varepsilon_{d}}}\geq k_{0}$, with $k_{0}=\frac{\omega }{c}$ being the free-space propagating wavevector, and $\varepsilon_{m}<0$ and $\varepsilon_{d}>0$ being the permittivities for the metal and dielectric media [34,35,36], respectively. Figure 1a,b show the widely known Kretschmann [37] and Otto [38] configurations, respectively, based on the attenuated total reflection (ATR) method. In these approaches, a transverse magnetic (TM) polarized light reaching the metallic surface from the prism has a wavevector component parallel to the surface, $k_{x}=k_{0}\sqrt{\varepsilon_{P}}\sin\theta$, which couples to the SPR mode under the wavevector matching condition

(1) $$k_{0}\sqrt{\varepsilon_{P}}\sin\theta =k_{0}\sqrt{\frac{\varepsilon_{m}\varepsilon_{d}}{\varepsilon_{m}+\varepsilon_{d}}},$$

where θ is the incidence angle and $\varepsilon_{P}$ is the permittivity of the prism. This latter condition can only be satisfied with $\varepsilon_{P}>\varepsilon_{d}$. The resonance angle, obtained from Equation (1) as $\theta_{\mathrm{SPR}}=\sin^{-1}\left(\frac{1}{\sqrt{\varepsilon_{P}}}\sqrt{\frac{\varepsilon_{m}\varepsilon_{d}}{\varepsilon_{m}+\varepsilon_{d}}}\right)$, is very sensitive to dielectric properties of the surrounding media, which is extensively exploited for plasmonic sensing and biosensing purposes [34,35,36]. In telecommunications applications, on the other hand, the Kretschmann setup is used for photodetection and optical signal processing through the excitation and detection of Bloch surface waves (BSWs) [39,40]. Despite the high performance of these structures, the need to use prism couplers for SPR excitation hampers on-chip integration. Figure 1c, on the other hand, uses diffraction to couple light to SPR modes through a diffraction grating. In this case, light is split into a series of beams with $k_{x}=k_{0}\sin\theta +mG$, where $G=\frac{2\pi }{\Lambda }$, with *m* and Λ being an integer and the period length of the grating, respectively. The wavevector matching condition in this case becomes

(2) $$k_{0}\sin\theta +\frac{2\pi }{\Lambda }m=k_{0}\sqrt{\frac{\varepsilon_{m}\varepsilon_{d}}{\varepsilon_{m}+\varepsilon_{d}}}.$$

Analogous to the prism coupler approach, these plasmonic grating structures have also been used for sensing and biosensing applications [41,42,43,44]. In telecommunications, their applications range from transmission lines to optical modulators [45,46,47,48,49,50]. In contrast to SPR approaches, LSPRs are excited by freely propagating light impinging on metallic nanoparticles or other finite structures. In this case, the harmonic oscillation of the electric field of light produces oscillation of the free-electron-density in the metal surface, analogous to a forced harmonic oscillator. The maximum dipolar amplitudes are observed when the frequency of light matches the natural frequency of the electron density, known as the plasmon frequency ($\omega_{p}$) [35]. These extremely localized and enhanced fields are commonly used for surface-enhanced-phenomena in plasmonic biosensing applications [51,52]. In the case of small and highly symmetric metallic nanoparticles, the electromagnetic response can be well-described through the quasi-static approximation [53]. In telecommunications applications, where LSPRs and SPRs are synergistically used [54] to convert free radiation into guided signals and vice versa, previous analytical expressions are useful for qualitative purposes, whereas proper theoretical analyses can only be made using specialized numerical methods [11,14,15].

## 3. Telecommunications Applications

Plasmonic resonances, hybrid electronic-photonic modes, allow for on-chip devices seamlessly integrating the exceptional data rates of optical signals with the extreme miniaturization features of nanoelectronic circuits [55,56,57,58,59]. In particular, plasmonic nanoantennas, i.e., nanoscale analogous of RF antennas, has become a recent trend of research due to their incredible ability to convert localized into propagating far-field electromagnetic waves and vice versa [11,12,13,14,15]. However, at these high frequency regimes light penetrates the metals and ohmic losses become substantially higher, i.e., metals cannot be considered as perfect electric conductors (PEC), drastically changing the way they are designed in comparison to their RF counterparts. Other applications for proper implementation of plasmonic nanocircuits include plasmonic waveguides, modulators, photodetectors, filters, resonators, and switches among others, which we also survey here [60,61].

### 3.1. Plasmonic Nanoantennas

Plasmonic waveguides, structures able to confine and enhance electromagnetic fields at subdiffraction limits, are used for optical signal transmission among different nanophotonic circuit components [62]. These nanostructures can be developed in several different ways like, for example, flat planar multilayer slabs, nanoparticle arranges, metal-coated-fibers, metallic nanowires, and metal gratings, among others [63,64,65,66,67,68,69,70,71,72,73,74]. Although these systems allow for ultra-low interconnection delays with subwavelength scale devices, practical applications are hindered by energy dissipation and crosstalk between adjacent plasmonic waveguides [14,75]. In particular, ohmic losses in metals at optical frequencies limit propagation lengths up to only some few micrometers, stimulating the search for other propagation mechanisms. Wireless information transfer at nanoscale has recently emerged as a promising alternative [76,77,78,79]. In this approach, communication performance is strongly dependent on the design of the corresponding nanolinks, i.e., the nanoemitters and nanoreceivers, for which RF antenna theory is being actively used [11,12,13,14,15]. We will review here some of the most recent approaches for highly efficient nanoantenna designs.

**Figure 2:**
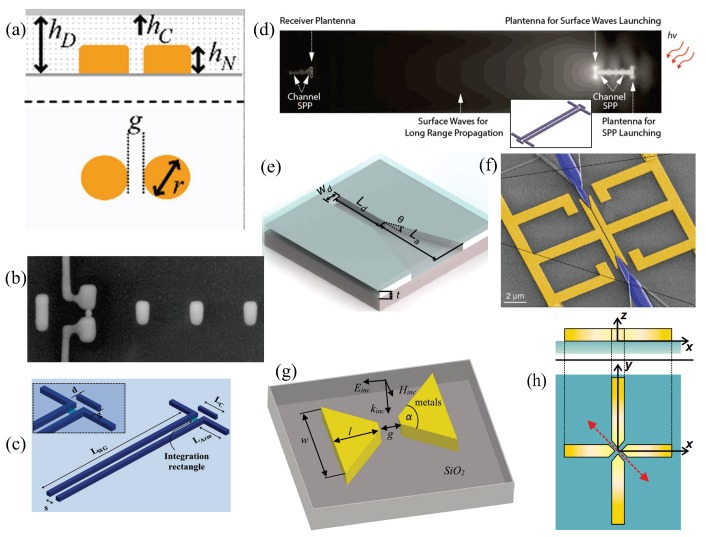
(**a**) Pictorial representation of a dipole nanoantenna, built by two nearby gold nanocylinders, embedded in an indium-tin-oxide (ITO) coated glass substrate. (**b**) A scanning electron microscope (SEM) image of a plasmonic Yagi–Uda nanoantenna composed by a reflector, feed element with kinked connectors and three directors on a glass substrate. (**c**) Schematic of a Plantenna design. (**d**) Illustration of a wireless communication nanolink. Plantenna receiver (left side) and transceiver (right side) are represented. (**e**) Schematic of a plasmonic horn nanoantenna, and its feeding waveguide, carved in a metallic film. This plasmonic platform is embedded between a substrate and a dielectric cladding layer. (**f**) False color SEM micrograph of a two-coupled low- and high-frequency nanoantenna forming a plasmonic phase-shifter (PPS) waveguide. (**g**) Schematic of a single bowtie nanoantenna. (**h**) Sketch of an asymmetric plasmonic cross nanoantenna. (**a**) Adapted with permission from Reference [85]; Copyright 2018 Springer Nature. (**b**) Used with permission from Reference [100]; Copyright 2020 Springer Nature. (**c**) Adapted with permission from Reference [79]; Copyright 2015 Springer Nature. (**d**) Adapted with permission from Reference [14]; Copyright 2017 American Chemical Society. (**e**) Used with permission from [102]; Copyright 2016 Springer Nature. (**f**) Adapted with permission from Reference [110]; Copyright 2019 Springer Nature. (**g**) Used with permission from Reference [114]; Copyright 2019 Springer Nature. (**h**) Used with permission from Reference [118]; Copyright 2009 American Physical Society.

The most simple nanoantenna one can imagine is a single or two-coupled metallic nanoscatterers (dipole nanoantenna) [80,81,82,83,84,85,86], as illustrated in Figure 2a. The scattering properties of these systems have been extensively investigated (from the numerical and experimental points of view) as a function of the nanoparticles geometry and the input impedance [82,85], demonstrating that it can be designed analogous to dipoles in RF domain [80,81,84]. These concepts have also opened up the possibility to design and develop optical nanocircuit elements analogous to radiation resistance, radiation efficiency, and conduction losses [83,87]. However, the lack of directionality hampers applications in optical signal transmission. An approach to circumvent this limitation uses plasmonic nanoparticle arrangements deposited over a properly designed multilayer substrate [88], which may result in a difficult and expensive mechanism. A recent alternative to control the directional radiation with nanoparticles, inspired by the well-known RF Yagi–Uda antenna design [89,90,91,92,93,94,95,96,97,98,99,100], considers an array of scatterers to achieve constructive interference of optical waves in one direction, whereas destructive in the opposite, as schematized in Figure 2b. This latter system efficiently links electron-based integrated computer chips to photon-based fiber networks for on-chip optical data transmission [100]. Importantly, these nanoantennas can be directly driven by optical or electrical signals [99,100]. Even though plasmonic Yagi–Uda nanoantennas show high directivity and ease of implementation, their performance can be severely limited due to their high sensitivity to the surrounding media [100]. In this regard, a waveguide-fed nanoscale analogous of the dipole aerial antenna was proposed [11] as a way to overcome the ohmic losses, when transmitting an optical signal, over several wavelengths at microscale level. Such a model was further improved [79] by placing a plasmonic nanoparticle adjacent to the dipole nanoantenna, as depicted in Figure 2c. Such a design hugely outperforms either the waveguide or dipole nanoantenna alternatives. In transmitting mode, the plasmonic nanoparticle works as a director, improving the directivity respect to their simplified dipole nanoantenna design [11]; whereas in the receiving mode, the field enhancement at the nanoparticle tips, enormously enhance the received signal for improved efficiency and longer distances. These properties led the researchers to develop a new plasmonic nanotransceiver [14] named Plantenna design, depicted in Figure 2d. These wireless optical data transfer/receiver links can be arranged with λ/2 separation in order to reach an efficient energy and data transfer up to millimeter distances [14]. Another waveguide-fed nanoscale analogue to a widely known RF model is the plasmonic horn nanoantenna [12,13,101,102,103,104], illustrated in Figure 2e. This concept exploits the high directivity, low reflection, and planar far-field radiation pattern properties of conventional RF horn antennas at nanoscale levels. These systems can be developed by horn-like nanostructured [12,13,101] or slotted [102] metallic films for nanoscale beam steering. In comparison to the Plantenna design, the tunable high-directivity of plasmonic horn nanoantennas make them ideal candidates to work in the transfer mode, while the ability of Plantenna to amplify the received signal to operate in the receiving mode. This idea was numerically shown in a recent work [15], demonstrating that a hybrid impedance-matched horn-Plantenna optical nanolink can greatly outperform other recent proposals in terms of efficiency and communication distance. These optical nanolinks have potential in the future 6G telecommunication networks, envisioned to work at terabits per second (Tbps) data rates [105,106,107], for which optical nanoantennas are of fundamental importance in the design and development of ultracompact and ultrafast optical nanocircuits [108]. To make this a reality, seamless integration among THz links, inter-/intra-chip nanoelectronic functions, and the existing fiber-optic infrastructure is of crucial interest to avoid communication-performance bottlenecks [4,15]. In this regard, plasmonic nanoantennas also work as field detectors/modulators to mediate among optical, THz, and RF fields [109,110].

Figure 2f shows a plasmonic nanoantenna design consisting of two metallic arms, forming a plasmonic slot waveguide, filled with a second-order nonlinear organic material [110]. These systems can be used to accurately measure the amplitude and phase of THz signals if included as a plasmonic phase-shifter in a Mach–Zehnder interferometer (MZI) configuration [110], where optical probes are converted into SPRs and back to Si waveguides. Other approaches include the bowtie nanoantenna [111,112,113], i.e., a dipole-type nanoantenna built by two nearby metallic nanotriangles or nanopyramids separated by a small gap [114], as illustrated in Figure 2g. This configuration produces a very large near-field enhancement, with its EM field concentrated in the gap region [115,116,117]. A modified version of this dipole consists in the use of plasmonic cross nanoantennas [118,119], i.e., two dipoles placed perpendicularly to each other, as depicted in Figure 2h. Its shape not only allows for light enhancement and confinement but also to control the wave polarization [120], with applications for high-speed optoelectronic devices [121] and photodetectors [122]. Other geometrical approaches including J-pole, mirrored J-pole, and Vee, among others, have been numerically and experimentally studied [123,124,125]. The ability of these plasmonic structures for bending of light at-will stimulated several new trends including metasurfaces, LIDAR antenna arrays, and integration with microwave photonics for the development of faster high-performance optical-wireless communication [126,127,128,129,130].

### 3.2. Plasmonic Modulators

Researchers are seeking for ultrafast wireless THz links able to seamlessly integrate optical-to-THz (O/T) data carriers and vice versa in order to cope with the ever-growing need for higher data rates in wireless communication networks [4]. In addition to the enormous potential of plasmonic nanoantennas for this purpose [110], discussed in the previous section, there are also other plasmonic nanostructures offering high-performance and compactness to fit a coded data into the photonic domain through coherent detection of amplitude, phase, or both [131,132,133]. In this section, we will review some of the most promising approaches for signal modulation by plasmonic devices.

**Figure 3:**
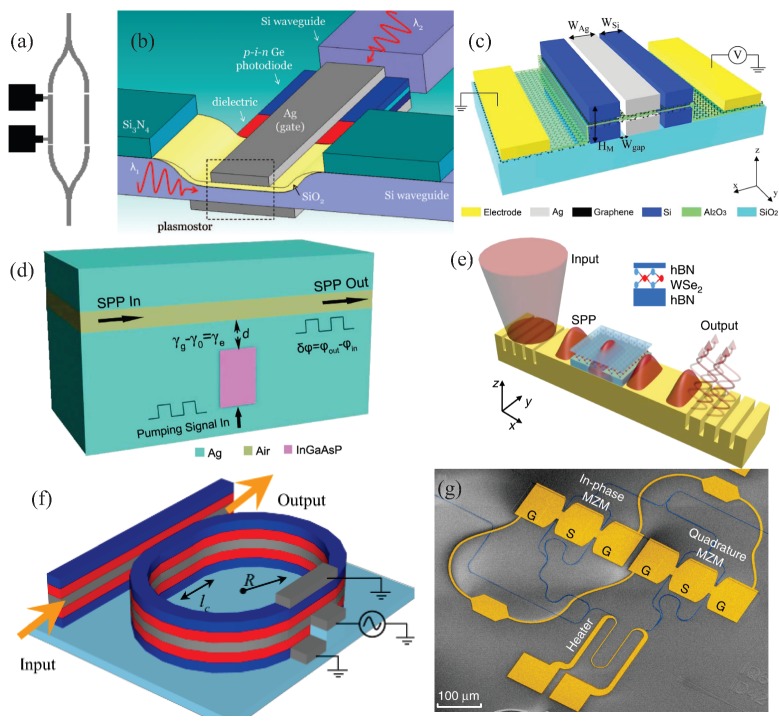
(**a**) Pictorial view of a Y-shaped Mach–Zender Modulator (MZM). (**b**) Schematic of an all-optical plasmonic metal-oxide-semiconductor (MOS) modulator. (**c**) Depiction of an insulator-metal-insulator graphene-based double-slots plasmonic waveguide modulator. (**d**) Illustrative representation of a plasmonic metal-insulator-metal (MIM) waveguide side-coupled to a plasmonic rectangular resonator. (**e**) Schematic of a two-dimensional hBN-WSe_2_-hBN (hexagonal Boron Nitride—Tungsten Diselenide—hexagonal Boron Nitride) semiconductor over a grating-coupler-based plasmonic waveguide for exciton-SPP coupling. (**f**) Representation of hybrid plasmonic waveguide laterally coupled to a plasmonic ring resonator. Both systems are built as a silicon-EOP-silver-EOP-silicon multilayer on SiO_2_ substrate. EOP—electro-optic polymers. (**g**) False color SEM micrograph of the two imbalanced high-speed plasmonic MZMs integrated into a SiP MZI. (**a**) Adapted with permission from Reference [134]; Copyright 2004 AIP Publishing. (**b**) Used with permission from Reference [137]; Copyright 2009 American Chemical Society. (**c**) Adapted with permission from Reference [141]; Copyright 2018 Springer Nature. (**d**) Adapted with permission from Reference [143]; Copyright 2016 Springer Nature. (**e**) Used with permission from [145]; Copyright 2019 Springer Nature. (**f**) Adapted with permission from Reference [146]; Copyright 2019 IEEE. (**g**) Used with permission from Reference [148]; Copyright 2019 Springer Nature.

Figure 3a shows an example of the Mach–Zender Modulator (MZM). This concept uses two gold stripe waveguides embedded in polymer, as depicted. One of the stripes is heated by electrical currents in order to introduce a phase-mismatch between the two MZM arms, which in turn changes the output amplitude. This modulation mechanism was successfully used for signal modulation at telecommunication wavelengths in the range of 1.51 μm to 1.62 μm [134]. Analogous structures have also been proposed recently [135,136]. The need for ultracompact silicon-compatible modulators, for on-chip all-optical and optoelectronic computational networks, has also stimulated other approaches. A metal-oxide-semiconductor (MOS) modulator, based on multimode interferometry in a plasmonic waveguide, exploiting the subwavelength strong SPR electromagnetic field enhancement for improved electro-optical nonlinearities has been proposed [137]. This plasmonic-based MOS modulator, named PlasMOStor, is schematized in Figure 3b. The PlasMOStor-based signal modulation is made by exploiting the fast modulation of accumulation conditions in the MOS capacitor, reaching modulation ratios around 10 dB. More recently, the unique optical properties of graphene [138,139,140] are also calling research attention to boost modulation performance, speeds, and optical bandwidths [141,142]. An approach for these graphene-based plasmonic modulators considers a capacitive graphene-insulator-graphene double layer in between two insulator-metal-insulator (IMI) platform [141], as depicted in Figure 3c. The chemical potential of graphene layers, working as capacitor and light absorber, is electrically controlled for efficient signal modulation.

Plasmonic waveguide modulation can also be reached by using a nearby nanoresonator filled with a gain medium (InGaAsP) [143]. A prototypical structure is illustrated in Figure 3d, consisting of a plasmonic metal-insulator-metal (MIM) waveguide, made by a silver-air-silver channel, with a side-coupled plasmonic rectangular resonator. In this approach, the cavity is electrically pumped to compensate the intrinsic ohmic losses of SPRs [144], also known as surface plasmon polaritons (SPPs), propagating in the waveguide. This waveguide-resonator system enables high-contrast modulation, allowing phase shifting up to 180°, with bandwidths up to 100 GHz, and speeds in the order of 0.2 ns. Figure 3e, on the other hand, illustrates a 2D semiconductor-plasmonic heterostructure for an ultra-low switching energy plasmonic modulator [145]. This latter structure couples an SPP mode, propagating along the plasmonic waveguide, with excitons in the two-dimensional hBN-WSe_2_-hBN (hexagonal Boron Nitride—Tungsten Diselenide—hexagonal Boron Nitride) semiconductor. The system can work in the linear and nonlinear regimes, through proper modulating of the pump and probe beams, reaching modulation bandwidths around 1.5 THz. Another recent proposal considers a hybrid plasmonic modulator, combining silicon and electro-optic polymers (EOP) with silver, built by a straight waveguide coupled to a ring resonator [146]. The system is developed, from bottom to top, as silicon-EOP-silver-EOP-silicon on SiO_2_ substrate, as depicted in Figure 3f. An externally applied voltage in the ring resonator, applied to change the EOP refractive index, is used for modulation of the plasmonic mode along the EOP/silver/EOP interfaces. An in-phase/quadrature (IQ) modulator [147], encoding information into the phase and amplitude of light, with attojoule per bit electrical energy consumption has been recently demonstrated [148]. The system, consisting of two imbalanced high-speed plasmonic MZMs integrated into a SiP Mach–Zehnder interferometer (MZI), is shown in Figure 3g. The phase delay in this platform can be adjusted either by a thermo-optic phase shifter (heater) or by tuning the wavelength, offering complex modulation up to 400 Gbps on a compact footprint.

A recent approach developed an Mach–Zehnder interferometer (MZI) all-plasmonic 116 Gbps electro-optical modulator from a single layer of gold using a substrate-independent process [149]. High-compactness, high-speed, and low-cost were reached through exploitation of the electro-optical Pockels effect [150,151] and the integration with a multicore optical fiber for SPR excitation [152] and polarization beam splitters [153,154,155,156]. An electro-optical modulator, based on a ring-resonator coupled to a buried low-loss silicon photonic waveguide, has also been recently proposed [157]. This latter proposal demonstrated an improved bandwidth and resilience to high temperature by replacing the conventionally used LiNbO_3_ (lithium niobate) for BaTiO_3_ (barium titanate) [158]. Other concepts include the use of hybrid plasmonic-polymer devices [47,159], liquid-crystal electro-optic plasmonic platforms [160,161], and carrier accumulation/epsilon-near-zero effect in transparent conductive oxides [162,163].

### 3.3. Plasmonic Filters, Switches, Routers, and Photodetectors

**Figure 4:**
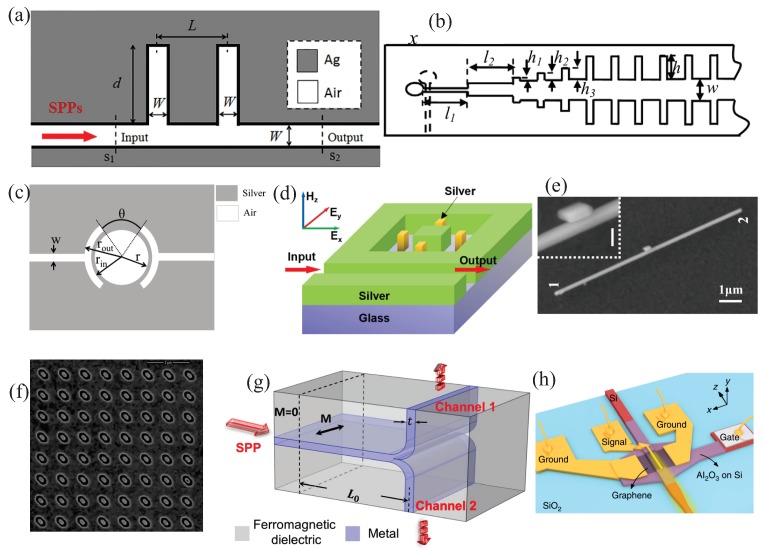
(**a**) Schematic of a Ag plasmonic waveguide coupled with two identical tooth-like cavities for band-stop filters. (**b**) Front view of the design of a microwave filter, consisting of two metallic gratings in opposite directions for improved confinement of SSPP waves. (**c**) Pictorial view of a symmetrical plasmonic ring-resonator-based filter. (**d**) Three-dimensional view of a nanoblock-loaded rectangular cavity. (**e**) SEM picture of a nanowire-nanoparticle plasmonic waveguide. (**f**) SEM micrograph of a two-dimensional chiral plasmonic metasurface. (**g**) Schematic of a switchable plasmonic router. (**h**) Illustrative representation of a hybrid silicon-graphene plasmonic waveguide photodetector. (**a**) and (**b**) were adapted with permission from Reference [171]; Copyright 2019 Elsevier. (**b**) Used with permission from Reference [172]; Copyright 2014 AIP Publishing. (**c**) Adapted with permission from Reference [184]; Copyright 2018 Elsevier. (**d**) Adapted with permission from Reference [185]; Copyright 2019 Elsevier. (**e**) Used with permission from [199]; Copyright 2019 Elsevier. (**f**) Adapted with permission from Reference [201]; Copyright 2019 Elsevier. (**g**) Used with permission from Reference [202]; Copyright 2018 Springer Nature. (**h**) Adapted with permission from Reference [210]; Copyright 2020 Springer Nature.

In addition to plasmonic waveguides, nanoantennas, and modulators, previously discussed, plasmonic filters are deserving attention for telecommunications applications, by virtue of their ability for frequency-selective absorption/transmission of optical signals, through proper design of metallic nanostructures [164,165,166]. Importantly, plasmonic notch filters inspired in tooth-based waveguides [167,168,169] have recently been developed at THz frequency range [170,171]. Figure 4a illustrates a plasmonic Ag cavity having two tooth-like cavities, filled with air, for rejection of wavelengths around 1 μm [171]. Previous approaches with metallic teeth-shape cavities on both sides, as illustrated in Figure 4b, have been demonstrated at microwave regime [172]. It is worth mentioning that these electromagnetic surface modes are named spoof surface plasmon polaritons (SSPP) as they mimic the optical SPR properties [173,174]. Although SSPPs are used in telecommunications for several applications like antennas [175,176,177] and transmission lines [178,179], they have shown an exceptional ability for highly efficient wavelength filtering [180,181]. Other filters’ approaches include hexagonal [182,183] and ring resonators [184], as the one illustrated in Figure 4c, enabling easy tuning of rejection-band at mid-infrared frequencies by simply varying the ring’s radius. In the case of hexagonal resonators, it can be easily coupled to a teeth-shape structure for single-mode filter applications [183]. Rectangular cavities, on the other hand, work as rejection-band filters [185] or wavelength demultiplexers [186]. Figure 4d schematically shows a rectangular cavity, with silver nanoblocks inside, for wavelength splitting and shifting [185]. Analogous platforms have been fed by a single input plasmonic waveguide and coupled to four output channels for wavelength mutiplexing/deplexing [187].

Plasmonic switches and routers are devices enabling selective propagation through different paths in a multiwaveguide system [188,189,190,191]. This selectivity allows for Boolean algebra [192], i.e., logical gates, and thus for optical computing with highly integrable and CMOS (Complementary Metal Oxide Semiconductor)-compatible devices [193,194,195,196]. Nonlinear optical phenomena, on the other hand, have also been recently demonstrated to be useful for plasmonic-based switching applications [197]. Of particular importance, symmetry-breaking features of plasmonic nanowires can also be used for polarization beam splitting, switching, and routing of light fields [198,199]. A SEM picture of a thick nanowire with adjacent nanoparticles is shown in Figure 4e. In this system, the output signal can be modulated/switched through the polarization angle of the input signals in port1 (1 in figure), port2 (2 in figure), or both. Chiral plasmonic structures [200], i.e., plasmonics systems whose opposite mirror images cannot be superimposed through symmetry operations, can also be used for routing of circularly polarized light (CPL) [201]. Figure 4f shows a SEM micrograph of a two-dimensional gold nanostructure used for polarization-based routing of the incident CPL. Magneto-optical (MO) properties of light have been used for externally controlled routing of SPP modes through two different paths [202], as depicted in Figure 4g. In this case, nanostructured plasmonic and MO materials is required. The intrinsic magnetization (M) of a ferromagnetic dielectric (MO material) can be alternated by an externally applied magnetic field in order to selectively route the SPP guided mode to travel along channel 1/channel 2 in the structure. Other proposals include hybrid photonic-plasmonic, Bragg-grating-based, and graphene-based router and switching devices [203,204,205]. Plasmonic photodetectors, on the other hand, enable fast and efficient detection and demodulation of optical signals [206,207]. In this context, plasmonic nanoantennas can also be used to downscale conventional semiconductor photodetectors—of high importance for THz and infrared detection [208,209]. A hybrid plasmonic silicon-graphene waveguide photodetector device [210], for light detection at 1.55 μm, is depicted in Figure 4h. These kinds of platforms allow broadband, low-footprint, and high-speed optical data reception, in addition to being CMOS compatible. Alternative graphene-based plasmonic photodetection devices have also been presented [211,212,213].

## 4. Outlook

**Figure 5:**
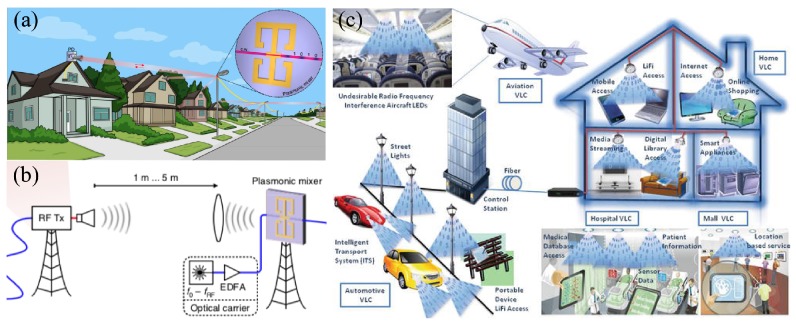
(**a**) Potential scenario of application for plasmonic-aided telecommunications in a neighborhood. (**b**) Schematic of an optical-wireless link. (**c**) Prospective scenarios of visible light communications applications, which can be boosted through the use of plasmonic features. (**a**) and (**b**) were adapted with permission from Reference [231]; Copyright 2018 Springer Nature. (**c**) Used with permission from Reference [232]; Copyright 2017 IntechOpen.

The unparalleled ability of plasmonic nanostructures for light confinement and enhancement has been shown promising for future data networks. Indeed, at-will guiding and beam steering of optical pulses at nanoscale levels are envisaged as a key element for 6G communications [4], where data rates in the order of Tbps—in addition to an increasing number of end devices—are expected [15]. To this end, a seamless interconnection of terahertz signals from fiber networks, wireless cells, and inter-/intra-chip nanoelectronic functions must be ensured. Based on recent developments discussed in this review, we may foresee plasmonic elements as integral parts of future telecommunication networks. Such developments would benefit not only from the ability of plasmonics for optical signals propagation, but also from plasmonic-aided photovoltaics for energy efficient devices [214]. Plasmonic reconfigurable intelligent surfaces [215,216], on the other hand, have potential applications in the development of revolutionary wireless technologies for beam bending at THz frequencies [217]. We also envisage the application of the Pancharatnam–Berry geometric phase, recently used for integrated millimeter-wave broadband CPL single-beam/multibeam antennas [218], for the development of analogous platforms at nanoscale dimensions [219,220]. Other concepts like MO and magnetoplasmonic effects are expected [221,222,223,224,225]. Moreover, ε-near-zero magnetoplasmonic heterostructures, previously used for biosensing applications, can also find applications in magnetoplasmonic waveguiding and routing at infrared regimes [226,227]. It is also worth mentioning the increasing interest in wireless visible light communication (LiFi) for low-latency machine-to-machine (car-to-car for instance) communication, for which plasmonics can also be used to improve the signal emission/reception efficiency [228,229,230]. A potential application scenario is illustrated in Figure 5. A plasmonic mixer for optical-to-wireless or wireless-to-optical conversion, located at lamp posts close to the houses in a residential area, is illustrated in Figure 5a. These mixers are expected to efficiently communicate with existing RF platforms [231], as illustrated in Figure 5b. The signal from the lamp post to houses can be transmitted via wireless visible light communication technology [232], which is expected to have higher transfer data rates and allowing for machine-type communication (as previously commented), as depicted in Figure 5c.

## References

[1] Wijayanto Y.N., Murata H., Okamura Y. (2013). Electrooptic Millimeter-Wave Lightwave Signal Converters Suspended to Gap-Embedded Patch Antennas on Low-*k* Dielectric Materials. IEEE J. Sel. Top. Quant. Elect..

[2] Leuthold J., Bonjour R., Salamin Y., Hoessbacher C., Heni W., Haffner C., Josten A., Baeuerle B., Ayata M., Messner A. Plasmonics for Communications. Proceedings of the 2018 Optical Fiber Communications Conference and Exposition (OFC).

[3] Thraskias C.A., Lallas E.N., Neumann N., Schares L., Offrein B.J., Henker R., Plettemeier D., Ellinger F., Leuthold J., Tomkos I. (2018). Survey of photonic and plasmonic interconnect technologies for intra-datacenter and high-performance computing communications. IEEE Commun. Surv. Tutor..

[4] Ummethala S., Harter T., Koehnle K., Muehlbrandt S., Kutuvantavida Y., Kemal J., Marin-Palomo P., Schaefer J., Tessmann A., Garlapati S.K. (2019). THz-to-Optical Conversion in Wireless Communications Using an Ultra-Broadband Plasmonic Modulator. Nat. Photon..

[5] Zúñiga D.J.C., Mafra S.B., Mejía-Salazar J.R., Montejo-Sánchez S., Fernandez E.M.G., Céspedes S. (2019). Visible Light V2V Cooperative Communication Under Environmental Interference. SBrT.

[6] Savaliya P.B., Gupta N., Dhawan A. (2019). Steerable plasmonic nanoantennas: Active steering of radiation patterns using phase change materials. Opt. Exp..

[7] Burla M., Salamin Y., Bonjour R., Abrecht F., Hoessbacher C., Haffner C., Heni W., Fedoryshyn Y., Werner D., Baeuerle B. Integrated photonic and plasmonic technologies for microwave signal processing enabling mm-wave and sub-THz wireless communication systems. Proceedings of the Broadband Access Communication Technologies XIII.

[8] Zia R., Schuller J.A., Chandran A., Brongersma M.L. (2006). Plasmonics: The Next Chip-Scale Technology. Mater. Today.

[9] Brongersma M.L., Shalaev V.M. (2010). The Case for Plasmonics. Science.

[10] Atabaki A.H., Moazeni S., Pavanello F., Gevorgyan H., Notaros J., Alloatti L., Wade M.T., Sun C., Kruger S.A., Meng H. (2018). Integrating photonics with silicon nanoelectronics for the next generation of systems on a chip. Nature.

[11] Alù A., Engheta N. (2010). Wireless at the Nanoscale: Optical Interconnects Using Matched Nanoantennas. Phys. Rev. Lett..

[12] Yang Y., Zhao D., Gong H., Li Q., Qiu M. (2014). Plasmonic Sectoral Horn Nanoantennas. Opt. Lett..

[13] Afridi A., Kocabaş Ş.E. (2016). Beam steering and impedance matching of plasmonic horn nanoantennas. Opt. Exp..

[14] Cohen M., Abulafia Y., Lev D., Lewis A., Shavit R., Zalevsky Z. (2017). Wireless Communication with Nanoplasmonic Data Carriers: Macroscale Propagation of Nanophotonic Plasmon Polaritons Probed by Near-Field Nanoimaging. Nano Lett..

[15] Alves A.A.C., Melo C.C., Siquieira J.J., Zanella F., Mejía-Salazar J.R., Cerqueira A.S. Plasmonic Nanoantennas for 6G Intra/Inter-Chip Optical-Wireless Communications. Proc. IEEE.

[16] Wei H., Pan D., Zhang S., Li Z., Li Q., Liu N., Xu H. (2018). Plasmon Waveguiding in Nanowires. Chem. Rev..

[17] Chen C., Mohr D.A., Choi H.K., Yoo D., Li M., Oh S.H. (2018). Waveguide-integrated compact plasmonic resonators for on-chip mid-infrared laser spectroscopy. Nano Lett..

[18] Zhu J., Xu Z., Xu W., Fu, D., Wei, D. (2018). Surface Plasmon Polariton Waveguide by Bottom and Top of Graphene. Plasmonics.

[19] Muench J.E., Ruocco A., Giambra M.A., Miseikis V., Zhang D., Wang J., Watson H.F.Y., Park G.C., Akhavan S., Midrio M. (2019). Waveguide-Integrated, Plasmonic Enhanced Graphene Photodetectors. Nano Lett..

[20] Li H., Chen B., Qin M., Wang L. (2020). Strong plasmon-exciton coupling in MIM waveguide-resonator systems with WS_2_ monolayer. Opt. Exp..

[21] Hoessbacher C., Josten A., Baeuerle B., Fedoryshyn Y., Hettrich H., Salamin Y., Heni W., Haffner C., Kaiser C., Schmid R. (2017). Plasmonic modulator with >170 GHz bandwidth demonstrated at 100 GBd NRZ. Opt. Exp..

[22] Burla M., Bonjour R., Salamin Y., Abrecht F., Hoessbacher C., Haffner C., Heni W., Fedoryshyn Y., Baeuerle B., Josten A. Plasmonics for Next-Generation Wireless Systems. Proceedings of the 2018 IEEE/MTT-S International Microwave Symposium—IMS.

[23] Li H.J., Wang L.L., Liu J.Q., Huang Z.R., Sun B., Zhai X. (2013). Investigation of the graphene based planar plasmonic filters. Appl. Phys. Lett..

[24] Zhuang T., Li S., Song G., Jiang P., Yu L. (2019). Tunable band-stop plasmonic waveguide filter with single-sided multiple-teeth-shaped structure. Phys. Scr..

[25] Schuck P.J., Fromm D.P., Sundaramurthy A., Kino G.S., Moerner W.E. (2005). Improving the mismatch between light and nanoscale objects with gold bowtie nanoantennas. Phys. Rev. Lett..

[26] Iizuka H., Fan S. (2013). Deep subwavelength plasmonic waveguide switch in double graphene layer structure. Appl. Phys. Lett..

[27] Nguyen D.M., Lee D., Rho J. (2017). Control of light absorbance using plasmonic grating based perfect absorber at visible and near-infrared wavelengths. Sci. Rep..

[28] Pitelet A., Mallet E., Ajib R., Lemaître C., Centeno E., Moreau A. (2018). Plasmonic enhancement of spatial dispersion effects in prism coupler experiments. Phys. Rev. B.

[29] Maier S.A. (2007). Plasmonics: Fundamentals and Applications.

[30] Paliwal A., Tomar M., Gupta V. (2019). Refractive Index Sensor Using Long-Range Surface Plasmon Resonance with Prism Coupler. Plasmonics.

[31] Chen J., Wang D., Xi J., Au L., Siekkinen A., Warsen A., Li Z.-Y., Zhang H., Xia Y., Li X. (2007). Immuno Gold Nanocages with Tailored Optical Properties for Targeted Photothermal Destruction of Cancer Cells. Nano Lett..

[32] Gobin A.M., Lee M.H., Halas N.J., James W.D., Drezek R.A., West J.L. (2007). Near-Infrared Resonant Nanoshells for Combined Optical Imaging and Photothermal Cancer Therapy. Nano Lett..

[33] Yoo K., Becker S.F., Silies M., Yu S., Lienau C., Park N. (2019). Steering second-harmonic radiation through local excitations of plasmon. Opt. Exp..

[34] Mejía-Salazar J.R., Camacho S.A., Constantino C.J.L., Oliveira O.N. (2018). New Trends in Plasmonic (Bio)Sensing. An. Acad. Bras. Ciênc..

[35] Mejía-Salazar J.R., Oliveira Jr O.N. (2018). Plasmonics Biosensing. Chem. Rev..

[36] Luo X., Tsai D., Gu M., Hong M. (2019). Extraordinary optical fields in nanostructures: From sub-diffraction-limited optics to sensing and energy conversion. Chem. Soc. Rev..

[37] Kretschmann E., Raether H. (1968). Radiative decay of non radiative surface plasmons excited by light. Z. Naturforsch A.

[38] Otto A. (1968). Excitation of nonradiative surface plasma waves in silver by the method of frustrated total reflection. Z. Physik.

[39] Lahijani B.V., Ghavifekr H.B., Dubey R., Kim M.-S., Vartiainen I., Roussey M., Herzig H.P. (2017). Experimental Demonstration of Critical Coupling of Whispering Gallery Mode Cavities on A Bloch Surface Wave Platform. Opt. Lett..

[40] Dubey R., Marchena M., Lahijani B.V., Kim M.-S., Pruneri V., Herzig H.P. (2018). Bloch Surface Waves Using Graphene Layers: An Approach Toward In-Plane Photodetectors. Appl. Sci..

[41] Diaz-Valencia B.F., Mejía-Salazar J.R., Oliveira O.N., Porras-Montenegro N., Albella P. (2017). Enhanced Transverse Magneto-Optical Kerr Effect in Magnetoplasmonic Crystals for the Design of Highly Sensitive Plasmonic (Bio)sensing Platforms. ACS Omega.

[42] Kamran M., Faryad M. (2018). Plasmonic Sensor Using a Combination of Grating and Prism Couplings. Plasmonics.

[43] Zhu J., Xu Z. (2019). Tunable temperature sensor based on an integrated plasmonic grating. Opt. Mat. Exp..

[44] Yan F., Li L., Wang R., Tian H., Liu J., Tian F., Zhang J. (2019). Ultrasensitive Tunable Terahertz Sensor with Graphene Plasmonic Grating. J. Light. Technol..

[45] Burgos S.P., Lee H.W., Feigenbaum E., Briggs R.M., Atwater H.A. (2014). Synthesis and Characterization of Plasmonic Resonant Guided Wave Networks. Nano Lett..

[46] Thackray B.D., Thomas P.A., Auton G.H., Rodriguez F.J., Marshall O.P., Kravets V.G., Grigorenko A.N. (2015). Super-Narrow, Extremely High Quality Collective Plasmon Resonances at Telecom Wavelengths and Their Application in a Hybrid Graphene-Plasmonic Modulator. Nano Lett..

[47] Emboras A., Hoessbacher C., Haffner C., Heni W., Koch U., Ma P., Fedoryshyn Y., Niegemann J., Hafner C., Leuthold J. (2015). Electrically Controlled Plasmonic Switches and Modulators. IEEE J. Select. Top. Quant. Electr..

[48] Thomas P.A., Auton G.H., Kundys D., Grigorenko A.N., Kravets V.G. (2017). Strong Coupling of Diffraction Coupled Plasmons and Optical Waveguide Modes in Gold Stripe-Dielectric Nanostructures at Telecom Wavelengths. Sci. Rep..

[49] Aghili S., Golmohammadi S., Amini A. (2018). Proposing An on/off Optical Router in Telecom Wavelength Using Plasmonic Tweezer. Opt. Commun..

[50] Anwar R.S., Ning H., Mao L. (2018). Recent Advancements In Surface Plasmon Polaritons-Plasmonics in Subwavelength Structures in Microwave and Terahertz Regimes. Dig. Commun. Net..

[51] Willets K., Van Duyne R. (2007). Localized Surface Plasmon Resonance Spectroscopy and Sensing. Annu. Rev. Phys. Chem..

[52] Jiang J., Wang X., Li S., Ding F., Li N., Meng S., Li R., Qi J., Liu Q., Liu G.L. (2018). Plasmonic nano-arrays for ultrasensitive bio-sensing. Nanophoton..

[53] Fröhlich H. (1949). Theory of Dielectrics: Dielectric Constant and Dielectric Loss.

[54] Sun Q., Oshikiri T., Yang J., Misawa H., Shi X., Ueno K., Gong Q. (2018). Manipulation of the dephasing time by strong coupling between localized and propagating surface plasmon modes. Nat. Commun..

[55] Torbatian Z., Asgari R. (2018). Plasmonic Physics of 2D Crystalline Materials. Appl. Sci..

[56] Kravets V.G., Kabashin A.V., Barnes W.L., Grigorenko A.N. (2018). Plasmonic Surface Lattice Resonances: A Review of Properties and Applications. Chem. Rev..

[57] Liu N., Liedl T. (2018). DNA-Assembled Advanced Plasmonic Architectures. Chem. Rev..

[58] Elder D., Johnson L., Tillack A., Robinson B.H., Haffner C., Heni W., Hoessbacher C., Fedoryshyn Y., Salamin Y., Baeuerle B. (2018). Multi-Scale Theory-Assisted Nano-Engineering of Plasmonic-Organic Hybrid Electro-Optic Device Performance. Proceedings of the Organic Photonic Materials and Devices XX.

[59] Ghideli M., Mascaretti B., Bricchi B.R., Zapelli A., Russo V., Casari C.S., Bassi A.L. (2018). Engineering Plasmonic Nanostructured Surfaces By Pulsed Laser Deposition. Appl. Surf. Sci..

[60] Sorger V.J., Oulton R.F., Ma R.M., Zhang X. (2012). Toward integrated plasmonic circuits. MRS Bull..

[61] Leuthold J., Hoessbacher C., Muehlbrandt S., Melikyan A., Kohl M., Koos C., Freude W., Dolores-Calzadilla V., Smit M., Suarez I. (2013). Plasmonic Communications: Light on a Wire. Opt. Phot. News.

[62] Fang Y., Sun M. (2015). Nanoplasmonic Waveguides: Towards Applications in Integrated Nanophotonic Circuits. Light. Sci. Appl..

[63] Krupin O., Berini P. (2019). Long-Range Surface Plasmon-Polariton Waveguide Biosensors for Human Cardiac Troponin I Detection. Sensors.

[64] Zhang Y., Xu Y., Tian C., Xu Q., Zhang X., Li Y., Zhang X., Han J., Zhang W. Corrugated Metal Surface With Pillars For Terahertz Surface Plasmon Polariton Waveguide Components. Proceedings of the SPIE 10623 International Conference on Optical Instruments and Technology: IRMMW-THz Technologies and Applications.

[65] Kim J.T., Choe J., Kim J., Dongjea S., Kim Y., Chung K. (2018). Graphene-based plasmonic waveguide devices for electronic-photonic integrated circuit. Opt. Laser Technol..

[66] Rifat A.A., Haider F., Ahmed R., Mahdiraji G.A., Mahamd Adikan F.R., Miroshnichenko A.E. (2018). Highly sensitive selectively coated photonic crystal fiber-based plasmonic sensor. Opt. Lett..

[67] Qu Y., Yuan J., Zhou X., Li F., Mei C., Yan B., Wu Q., Wang K., Sang X., Long K. (2019). A V-shape photonic crystal fiber polarization filter based on surface plasmon resonance effect. Opt. Commun..

[68] Ansell D., Radko I., Han Z., Rodriguez F.J., Bozhevolnyi S.I., Grigorenko A.N. (2015). Hybrid Graphene Plasmonic Waveguide Modulators. Nat. Commun..

[69] Colanduoni J., Nikolov D., Xu H. (2016). Multi-mode Hybrid Plasmonic Waveguides with Enhanced Confinement and Propagation. Plasmonics.

[70] Dabos G., Manolis A., Tsiokos D., Ketzaki D., Chatzianagnostou E., Markey L., Rusakov D., Weeber J.-C., Dereux A., Giesecke A.-L. (2018). Aluminum plasmonic waveguides co-integrated with Si_3_N_4_ photonics using CMOS processes. Sci. Rep..

[71] Zheng K., Yuan Y., He J., Gu G., Zhang F., Chen Y., Song J., Qu J. (2019). Ultra-High Light Confinement and Ultra-Long Propagation Distance Design for Integratable Optical Chips Based on Plasmonic Technology. Nanoscale.

[72] Roy B., Majumder S., Chakraborty R. (2020). Design Of Low Loss Surface Plasmon Polariton Waveguide and Its Use As Hybrid Tamm Sensor with Improved Sensitivity. Opt. Eng..

[73] Esmaeilzadeh H., Arzi E., Mozafari M., Hassani A. (2012). A Broadband Optical Fiber Based Inline Polarizer for Telecom Wavelength Range. Sens. Act. A.

[74] Liu F., Albert J. (2019). 40 Ghz-Rate All-Optical Cross-Modulation of Core-Guided Near Infrared Light in Single Mode Fiber By Surface Plasmons on Gold-Coated Tilted Fiber Bragg Gratings. APL Photon..

[75] Conway J.A., Sahni S., Szkopek T. (2007). Plasmonic interconnects versus conventional interconnects: A comparison of latency, crosstalk and energy costs. Opt. Exp..

[76] Liu Z., Boltasseva A., Pedersen R.H., Bakker R., Kildishev A.V., Drachev V.P., Vladimir M., Shalaev V. (2008). Plasmonic Nanoantenna Arrays for the Visible. Metamat.

[77] Ni X., Emani N.K., Kildishev A.V., Boltasseva A., Shalaev V.M. (2012). Broadband Light Bending with Plasmonic Nanoantennas. Science.

[78] Patel S.K., Argyropoulos C. (2015). Plasmonic Nanoantennas: Enhancing Light-Matter Interactions at the Nanoscale. EPJ Appl. Metamat.

[79] Cohen M., Shavit R., Zalevsky Z. (2015). Enabling High Efficiency Nanoplasmonics with Novel Nanoantenna Architectures. Sci. Rep..

[80] Alú A., Engheta N. (2008). Tuning The Scattering Response of Optical Nanoantennas with Nanocircuit Loads. Nat. Phot..

[81] Alú A., Engheta N. (2008). Input Impedance, Nanocircuit Loading, and Radiation Tuning of Optical Nanoantennas. Phys. Rev. Lett..

[82] Arquer F.P.G., Volski V., Verellen N., Vandenbosch G.A.E., Moshchalkov V.V. (2011). Engineering the Input Impedance of Optical Nano Dipole Antennas: Materials, Geometry and Excitation Effect. IEEE Trans. Ant. Prop..

[83] Nafari M., Jornet J.M. (2017). Modeling and Performance Analysis of Metallic Plasmonic Nano-Antennas for Wireless Optical Communication in Nanonetworks. IEEE Access.

[84] Greffet J.-J. (2017). World Scientific Handbook of Metamaterials and Plasmonics.

[85] Drachev V.P., Kildishev A.V., Borneman J.D., Chen K.-P., Shalaev V.M., Yamnitskiy K., Norwood R.A., Peyghambarian N., Marder S.R., Padilha L.A. (2018). Engineered nonlinear materials using gold nanoantenna array. Sci. Rep..

[86] Zhang C., Hugonin J.P., Coutrot A.L., Sauvan C., Marquier F., Greffet J.J. (2019). Antenna surface plasmon emission by inelastic tunneling. Nat. Commun..

[87] Engheta N. (2007). Circuits with Light at Nanoscale: Optical Nanocircuits Inspired by Metamaterials. Science.

[88] Ghadarghadr S., Hao Z., Mosallaei H. (2009). Plasmonic array nanoantennas on layered substrates: Modeling and radiation characteristics. Opt. Exp..

[89] Kosako T., Kadoya Y., Hofmann H.F. (2010). Directional control of light by a nano-optical Yagi-Uda antenna. Nat. Phot..

[90] Ahmadi A., Mosallaei H. (2010). Plasmonic Nanoloop Array Antenna. Opt. Lett..

[91] Stout B., Devilez A., Rolly B., Bonod N. (2011). Multipole methods for nanoantennas design: Applications to Yagi-Uda configurations. J. Opt. Soc. Am. B.

[92] Dregely D., Taubert R., Dorfmüller J., Vogelgesang R., Klaus K., Giessen H. (2011). 3D Optical Yagi-Uda Nanoantenna Array. Nat. Commun..

[93] Maksymov I.S., Staude I., Miroshnichenko A.E., Kivshar Y.S. (2012). Optical Yagi-Uda Nanoantennas. Nanophotonics.

[94] Liu Y.G., Choy W.C.H., Sha W.E.I., Chew W.C. (2012). Unidirectional And Wavelength-Selective Photonic Sphere-Array Nanoantennas. Opt. Lett..

[95] Kim J., Roh Y.-G., Cheon S., Choe J.-H., Lee J., Lee J., Jeong H., Kim U.J., Park Y., Song I.Y. (2014). Babinet-Inverted Optical Yagi-Uda Antenna for Unidirectional Radiation to Free Space. Nano Lett..

[96] Helmy F.E., Hussein M., Hameed M.F.O., Shaker A., El-Adawy M., Obayya S.A. Optimal design of yagi-uda nanoantennas based on elliptical shaped elements. Proceedings of the SPIE 10672, Nanophotonics VII, 106722G.

[97] Ho J., Fu Y.H., Dong Z., Paniagua-Dominguez R., Koay E.H.H., Yu Y.F., Valuckas V., Kuznetsov A.I., Yang J.K.W. (2018). Highly Directive Hybrid Metal-Dielectric Yagi-Uda Nanoantennas. ACS Nano.

[98] Rieger W., Heremans J.J., Ruan H., Kang Y., Claus R. (2018). Yagi-Uda nanoantenna enhanced metal-semiconductor-metal photodetector. Appl. Phys. Lett..

[99] Helmy F.E., Hussein M., Hameed M.F.O., Shaker A., El-Adawy M., Obayya S.S.A. Metallo-Dielectric Yagi-Uda Nanoantennas Based on Rectangular Shaped Elements. Proceedings of the Physics and Simulation of Optoelectronic Devices XXVII.

[100] Kullock R., Ochs M., Grimm P., Emmerling M., Hecht B. (2020). Electrically-Driven Yagi-Uda Antennas for Light. Nat. Commun..

[101] Ramaccia D., Bilotti F., Toscano A., Massaro A. (2011). Efficient and Wideband Horn Nanoantenna. Opt. Lett..

[102] Yang Y., Li Q., Qiu M. (2016). Broadband nanophotonic wireless links and networks using on-chip integrated plasmonic antennas. Sci. Rep..

[103] Chen S., Gordon R. (2018). An Analytic Approach to Nanofocusing with Pyramidal Horn Antennas. Plasmonics.

[104] Nourmohammadi A., Nikoufard M. (2019). Ultra-Wideband Photonic Hybrid Plasmonic Horn Nanoantenna with SOI Configuration. Silicon.

[105] David K., Elmirghani J., Haas H., You X.-H. (2019). Defining 6G: Challenges and Opportunities. IEEE Veh. Technol. Mag..

[106] Dang S., Amin O., Shihada B., Alouini M.-S. (2020). What should 6G be?. Nat. Electr..

[107] Zhang Z., Xiao Y., Ma Z., Xiao M., Ding Z., Lei X., Karagiannidis G.K., Fan P. (2019). 6g wireless networks: Vision, requirements, architecture, and key technologies. IEEE Veh. Technol. Mag..

[108] Davis T.J., Gómez D.E., Roberts A. (2016). Plasmonic Circuits for Manipulating Optical Information. Nanophotonics.

[109] Salamin Y., Heni W., Haffner C., Fedoryshyn Y., Hoessbacher C., Bonjour R., Zahner M., Hillerkuss D., Leuchtmann P., Elder D.L. (2015). Direct Conversion of Free Space Millimeter Waves to Optical Domain by Plasmonic Modulator Antenna. Nano Lett..

[110] Salamin Y., Benea-Chelmus I.C., Fedoryshyn Y., Heni W., Elder D.L., Dalton L.R., Faist J., Leuthold J. (2019). Compact and Ultra-Efficient Broadband Plasmonic Terahertz Field Detector. Nat. Commun..

[111] Lallas E. (2019). Key Roles of Plasmonics in Wireless THz Nanocommunications—A Survey. Appl. Sci..

[112] He X., Xiao G., Liu F., Lin F., Shi W. (2019). Flexible properties of THz graphene bowtie metamaterials structures. Opt. Mater. Exp..

[113] Khoshdel V., Shokooh-Saremi M. (2019). Increased Electric Field Enhancement and Broad Wavelength Tunability by Plasmonic Bow-tie Nano-antenna Based on Fractal Geometry with Grid. Photon. Nanostruct. Fundam. Appl..

[114] Morshed M., Li Z., Olbricht B.C., Fu L., Haque A., Li L., Rifat A.A., Rahmani M., Miroshnichenko A.E., Hattori H.T. (2019). High Fluence Chromium and Tungsten Bowtie Nano-antennas. Sci. Rep..

[115] Chen H., Bhuiya A.M., Liu R., Wasserman D.M., Toussaint K.C. (2014). Design, Fabrication, and Characterization of Near-IR Gold Bowtie Nanoantenna Arrays. J. Phys. Chem. C.

[116] Hren M., Campbell C., Casey A., Triplett G. Simulation and design of plasmonic gold bowtie nanoantennas. Proceedings of the SPIE 10891 Nanoscale Imaging, Sensing, and Actuation for Biomedical Applications XVI.

[117] Campbell C., Casey A., Hren M., Drobitch J., Triplett G. A comparison of simulated and fabricated gold bowtie nanoantennas for molecular fingerprinting. Proceedings of the SPIE 10891, Nanoscale Imaging, Sensing, and Actuation for Biomedical Applications XVI.

[118] Biagioni P., Savoini M., Huang J.-S., Duò L., Finazzi M., Hecht B. (2009). Near-Field Polarization Shaping by a Near-Resonant Plasmonic Cross Antenna. Phys. Rev. B.

[119] Klaer P., Razinskas G., Lehr M., Krewer K., Schertz F., Wu X.-F., Hecht B., Schönhense G., Elmers H.J. (2015). Robustness of Plasmonic Angular Momentum Confinement in Cross Resonant Optical Antennas. Appl. Phys. Lett..

[120] Biagioni P., Huang J.S., Duo L., Finazzi M., Hecht B. (2009). Cross Resonant Optical Antenna. Phys. Rev. Lett..

[121] Qin Y., Xiong X.Y., Wei E.I., Jiang L.J. (2018). Electrically tunable polarizer based on graphene-loaded plasmonic cross antenna. J. Phys. Cond. Matt..

[122] Yang A., Yang K., Zhou L., Li J., Tan X., Liu H., Song H., Tang J., Liu F., Yi F. (2017). Split-cross antenna based narrowband mid-infrared absorber for sensing applications. Opt. Commun..

[123] James T.D., Teo Z.Q., Gomez D.E., Davis T.J., Roberts A. (2013). The plasmonic J-pole antenna. Appl. Phys. Lett..

[124] James T.D., Davis T.J., Roberts A. (2014). Optical investigation of the J-pole and Vee antenna families. Opt. Exp..

[125] James T.D., Cadusch J.J., Earl S.K., Panchenko E., Mulvaney P., Davis T.J., Roberts A. Nanometers to centimeters: Novel optical nano-antennas, with an eye to scaled production. Proceedings of the Photonic and Phononic Properties of Engineered Nanostructures VI.

[126] Bonjour R., Burla M., Abrecht F.C., Welschen S., Hoessbacher C., Heni W., Gebrewold S.A., Baeuerle B., Josten A., Josten A. Plasmonic phased array feeder enabling symbol-by-symbol mm-wave beam steering at 60 GHz. Proceedings of the 2016 IEEE International Topical Meeting on Microwave Photonics (MWP).

[127] Marpaung D., Yao J., Capmany J. (2019). Integrated microwave photonics. Nat. Photon..

[128] Hosseininejad S.E., Rouhi K., Neshat M., Cabellos-Aparicio A., Abadal S., Alarcón E. (2019). Digital Metasurface Based on Graphene: An Application to Beam Steering in Terahertz Plasmonic Antennas. IEEE Trans. Nanotechnol..

[129] Lesina A.C., Goodwill D., Bernier E., Ramunno L., Berini P. (2019). Optical phased arrays for LIDAR: Beam steering via tunable plasmonic metasurfaces. arXiv.

[130] Khodadadi M., Nozhat N., Moshiri S.M.M. (2020). Analytic approach to design a wideband hybrid plasmonic nano-antenna based on the new director. Opt. Exp..

[131] Kumar A., Yu S.F., Li X. (2009). Design and analysis of a surface plasmon polariton modulator using the electro-optic effect. Appl. Opt..

[132] Koch U., Hoessbacher C., Niegemann J., Hafner C., Leuthold J. (2016). Digital plasmonic absorption modulator exploiting epsilon-near-zero in transparent conducting oxides. IEEE Photon. J..

[133] Babicheva V.E., Malureanu R., Lavrinenko A.V. (2013). Plasmonic finite-thickness metal-semiconductor-metal waveguide as ultra-compact modulator. Photon. Nanostruct. Fundam. Appl..

[134] Nikolajsen T., Leosson K., Bozhevolnyi S.I. (2004). Surface plasmon polariton based modulators and switches operating at telecom wavelengths. Appl. Phys. Lett..

[135] Melikyan A., Alloatti L., Muslija A., Hillerkuss D., Schindler P.C., Li J., Palmer R., Korn D., Muehlbrandt S., Muehlbrandt D.V. (2014). High-speed plasmonic phase modulators. Nat. Photon..

[136] Messner A., Haffner C., Heni W., Koch U., Leuthold J. (2018). Pockels-Effect Materials for Plasmonic Modulators. Novel Optical Materials and Applications.

[137] Dionne J.A., Diest K., Sweatlock L.A., Atwater H.A. (2009). PlasMOStor: A Metal-Oxide-Si Field Effect Plasmonic Modulator. Nano Lett..

[138] Low T., Avouris P. (2014). Graphene Plasmonics for Terahertz to Mid-Infrared Applications. ACS Nano.

[139] Fan Y., Shen N.-H., Zhang F., Zhao Q., Wei Z., Zhang P., Dong J., Fu Q., Li H., Soukoulis C.M. (2018). Graphene Photoexcited Graphene Metasurfaces: Significantly Enhanced and Tunable Magnetic Resonances. ACS Photon..

[140] Fan Y., Shen N.-H., Zhang F., Zhao Q., Wu H., Fu Q., Wei Z., Li H., Soukoulis C.M. (2019). Graphene Plasmonics: A Platform for 2D Optics. Adv. Opt. Mater..

[141] Hao R., Ye Z., Gu Y., Peng X., Chen H., Li E. (2018). Large Modulation Capacity in Graphene-Based Slot Modulators by Enhanced Hybrid Plasmonic Effects. Sci. Rep..

[142] Hao R., Ye Z., Peng X., Gu Y., Jiao J.Y., Zhu H., Sha W.E.I., Li E. (2018). Highly Efficient Graphene-Based Optical Modulator With Edge Plasmonic Effect. IEEE Photon. J..

[143] Im S.J., Ho G.S., Yang D.J., Hao Z.H., Zhou L., Kim N.C., Kim I.G., Wang Q.Q. (2016). Plasmonic Phase Modulator Based on Novel Loss-Overcompensated Coupling Between Nanoresonator and Waveguide. Sci. Rep..

[144] West P.R., Ishii S., Naik G.V., Emani N.K., Shalaev V.M., Boltasseva A. (2010). Searching for better plasmonic materials. Laser Photon. Rev..

[145] Klein M., Badada B.H., Binder R., Alfrey A., McKie M., Koehler M.R., Mandrus D.G., Taniguchi T., Watanabe K., LeRoy B.J. (2019). 2D Semiconductor Nonlinear Plasmonic Modulators. Nat. Commun..

[146] Sherif S.M., Elsayed M., Shahada L., Swillam M.A. (2019). Sub-Femtojoule Hybrid Plasmonic Optical Modulator. IEEE Photon. J..

[147] Haffner C., Heni W., Fedoryshyn Y., Baeuerle B., Josten A., Salamin Y., Bonjour R., Hoessbacher C., Emboras A., Elder D.L. Ultra-Compact Plasmonic IQ-Modulator. European Conference on Optical Communication. Proceedings of the 2015 European Conference on Optical Communication (ECOC).

[148] Heni W., Fedoryshyn Y., Baeuerle B., Josten A., Hoessbacher C.B., Messner A., Leuthold J. (2019). Plasmonic IQ Modulators with Attojoule Per Bit Electrical Energy Consumption. Nat. Commun..

[149] Ayata M., Fedoryshyn Y., Heni W., Baeuerle B., Josten A., Zahner M., Koch U., Salamin Y., Hoessbacher C., Haffner C. (2017). High-speed plasmonic modulator in a single metal layer. Science.

[150] Heni W., Haffner C., Elder D.L., Tillack A.F., Fedoryshyn Y., Cottier R., Salamin Y., Hoessbacher C., Koch U., Cheng B. (2017). Nonlinearities of organic electro-optic materials in nanoscale slots and implications for the optimum modulator design. Opt. Exp..

[151] Heni W., Kutuvantavida Y., Haffner C., Zwickel H., Kieninger C., Wolf S., Lauermann M., Fedoryshyn Y., Tillack A.F., Johnson L.E. (2017). Silicon-Organic and Plasmonic-Organic Hybrid Photonics. ACS Photon..

[152] Burla M., Hoessbacher C., Heni W., Haffner C., Fedoryshyn Y., Werner D., Leuthold J. (2018). 500 GHz plasmonic Mach-Zehnder modulator enabling sub-THz microwave photonics. APL Photon..

[153] Fan Z., Li S., Liu Q., Li J., Xie Y. (2015). Plasmonic Polarization Beam Splitter Based on Dual-Core Photonic Crystal Fiber. Plasmonics.

[154] Dou C., Jing X., Li S., Wu J., Wang Q. (2018). A compact and low-loss polarization splitter based on dual-core photonic crystal fiber. Opt. Quant. Electr..

[155] Zhao X.T., Hua L., Xiong Q., Jiang G.H., Cheng J.R. (2019). Ultra-short and broadband polarization splitter based on PCF and metal surface plasmons resonance. Opt. Quant. Electr..

[156] Lou J., Cheng T., Li S. (2019). Ultra-short polarization beam splitter with square lattice and gold film based on dual-core photonic crystal fiber. Optik.

[157] Haffner C., Chelladurai D., Fedoryshyn Y., Josten A., Baeuerle B., Heni W., Leuthold J. (2018). Low-loss plasmon-assisted electro-optic modulator. Nature.

[158] Messner A., Eltes F., Ma P., Abel S., Baeuerle B., Josten A., Leuthold J. (2019). Plasmonic Ferroelectric Modulators. J. Light. Technol..

[159] Zografopoulos D.C., Swillam M., Beccherelli R. (2016). Hybrid Plasmonic Modulators and Filters Based on Electromagnetically Induced Transparency. IEEE Photon. Technol. Lett..

[160] Çetin A.E., Yanik A.A., Mertiri A., Erramilli S., Müstecaplıoğlu Ŏ.E., Altug H. (2012). Field-effect active plasmonics for ultracompact electro-optic switching. Appl. Phys. Lett..

[161] Zografopoulos D.C., Beccherelli R. (2013). Design of a vertically coupled liquid-crystal long-range plasmonic optical switch. Appl. Phys. Lett..

[162] Feigenbaum E., Diest K., Atwater H.A. (2010). Unity-Order Index Change in Transparent Conducting Oxides at Visible Frequencies. Nano Lett..

[163] Sinatkas G., Pitilakis A., Zografopoulos D.C., Beccherelli R., Kriezis E.E. (2017). Transparent conducting oxide electro-optic modulators on silicon platforms: A comprehensive study based on the drift-diffusion semiconductor model. J. Appl. Phys..

[164] Lai W., Wen K., Lin J., Guo Z., Hu Q., Fang Y. (2018). Plasmonic filter and sensor based on a subwavelength end-coupled hexagonal resonator. Appl. Opt..

[165] Janković N., Cselyuszka N. (2019). High-Resolution Plasmonic Filter and Refractive Index Sensor Based on Perturbed Square Cavity with Slits and Orthogonal Feeding Scheme. Plasmonics.

[166] Fleischman D., Fountaine K.T., Bukowsky C.R., Tagliabue G., Sweatlock L.A., Atwater H.A. (2019). High Spectral Resolution Plasmonic Color Filters with Subwavelength Dimensions. ACS Photon..

[167] Lin X.S., Huang X.G. (2008). Tooth-shaped plasmonic waveguide filters with nanometeric sizes. Opt. Lett..

[168] Tao J., Huang X.G., Lin X., Zhang Q., Jin X. (2009). A narrow-band subwavelength plasmonic waveguide filter with asymmetrical multiple-teeth-shaped structure. Opt. Exp..

[169] Feng Y., Liu Y., Wang X., Dong D., Shi Y., Tang L. (2018). Tunable multichannel plasmonic filter based on a single graphene sheet on a Fibonacci quasiperiodic structure. Plasmonics.

[170] Moazami A., Hashemi M., Shirazi N.C. (2019). High Efficiency Tunable Graphene-Based Plasmonic Filter in the THz Frequency Range. Plasmonics.

[171] Li H., Jiao R.-Z. (2019). Plasmonic band-stop filters based on tooth structure. Opt. Commun..

[172] Gao X., Zhou L., Liao Z., Ma H.F., Cui T.J. (2014). An ultra-wideband surface plasmonic filter in microwave frequency. Appl. Phys. Lett..

[173] Burla M., Bonjour R., Salamin Y., Haffner C., Heni W., Hoessbacher C., Leuthold J. (2016). Microwave plasmonics: A novel platform for RF photonics. IEEE Int. Top. Meet. Microwave Photon..

[174] Guo Y.-J., Xu K.D., Deng X. (2019). Tunable enhanced sensing of ferrite film using meander-shaped spoof surface plasmon polariton waveguide. Appl. Phys. Exp..

[175] Zhuang K., Geng J., Wang K., Zhou H., Liang Y., Liang X., Zhu W., Jin R., Ma W. (2019). Pattern Reconfigurable Antenna Applying Spoof Surface Plasmon Polaritons for Wide Angle Beam Steering. IEEE Access.

[176] Liu L., Chen M., Yin X. (2020). Single-Layer High Gain Endfire Antenna Based on Spoof Surface Plasmon Polaritons. IEEE Access.

[177] Han Y., Gong S., Wang J., Li Y., Fan Y., Zhang J., Qu S. (2020). Shared-Aperture Antennas Based on Even- and Odd-Mode Spoof Surface Plasmon Polaritons. IEEE Trans. Antennas Propag..

[178] Becker S., Fip T., Rahm M. (2020). Routing of strongly confined terahertz spoof surface plasmon polaritons on metasurfaces along straight and curved pathways with subwavelength width. Opt. Exp..

[179] Kianinejad A., Chen Z.N., Qiu C.-W. (2016). Low-loss spoof surface plasmon slow-wave transmission lines with compact transition and high isolation. IEEE Trans. Microw. Theory Technol..

[180] Zhao H., Zhou P., Xu Z., Li S., Yang M., Liu L., Yin X. (2020). Tri-Band Band-Pass Filter Based on Multi-Mode Spoof Surface Plasmon Polaritons. IEEE Access.

[181] Yang Z., Zhang B., Chen W., Yang T. (2019). Rejection of Spoof SPPs Using the Second Resonant Mode of Vertical Split-Ring Resonator. IEEE Microw. Wirel. Compon. Lett..

[182] Lu H., Liu X., Mao D., Wang L., Gong Y. (2010). Tunable band-pass plasmonic waveguide filters with nanodisk resonators. Opt. Exp..

[183] Khani S., Danaie M., Rezaei P. (2019). Design of a single-mode plasmonic bandpass filter using a hexagonal resonator coupled to graded-stub waveguides. Plasmonics.

[184] Khani S., Danaie M., Rezaei P. (2018). Realization of single-mode plasmonic bandpass filters using improved nanodisk resonators. Opt. Commun..

[185] Shi L., He J., Tan C., Liu Y., Hu J., Wu X., Chen M., Zhang X., Zhan S. (2019). Plasmonic filter with highly selective wavelength in a fixed dimension based on the loaded rectangular ring cavity. Opt. Commun..

[186] Zhang Z., Yang J., He X., Han Y., Zhang J., Huang J., Chen D. (2018). Plasmonic filter and demultiplexer based on square ring resonator. Appl. Sci..

[187] Reza M.P., Granpayeh N., Hosseini S.P., Rahimzadegan A. (2019). Ultracompact double tunable two-channel plasmonic filter and 4-channel multi/demultiplexer design based on aperture-coupled plasmonic slot cavity. Opt. Commun..

[188] Fang Y., Li Z., Huang Y., Zhang S., Nordlander P., Halas N.J., Xu H. (2010). Branched silver nanowires as controllable plasmon routers. Nano Lett..

[189] David T., Gómez D., Eftekhari F. (2014). All-optical modulation and switching by a metamaterial of plasmonic circuits. Opt. Lett..

[190] Saito K., Tatsuma T. (2015). Asymmetric Three-Way Plasmonic Color Routers. Adv. Opt. Mater..

[191] Chelladurai D., Doderer M., Koch U., Fedoryshyn Y., Haffner C., Leuthold J. (2019). Low-loss hybrid plasmonic coupler. Opt. Exp..

[192] Gao L., Chen L., Wei H., Xu H. (2018). Lithographically fabricated gold nanowire waveguides for plasmonic routers and logic gates. Nanoscale.

[193] Wei H., Li Z., Tian X. (2011). Quantum dot-based local field imaging reveals plasmon-based interferometric logic in silver nanowire networks. Nano Lett..

[194] Zhang X., Ma Z., Luo R., Gu Y., Meng C., Wu X., Gong Q., Tong L. (2012). Single-nanowire surface plasmon gratings. Nanotechnology.

[195] Guo X., Ma Y., Wang Y. (2013). Nanowire plasmonic waveguides, circuits and devices. Laser Photon. Rev..

[196] Heni W., Hoessbacher C., Haffner C., Fedoryshyn Y., Baeuerle B., Josten A., Hillerkuss D., Salamin Y., Bonjour R., Melikyan A. (2015). High speed plasmonic modulator array enabling dense optical interconnect solutions. Opt. Exp..

[197] Gubin M.Y., Leksin A.Y., Shesterikov A.V., Volkov V.S., Prokhorov A.V. (2020). Nonlinear plasmonic switching in graphene-based stub nanoresonator loaded with core-shell nanowire. Appl. Surf. Sci..

[198] Yang L., Li P., Li Z. (2019). Plasmonic polarization beam splitting based on single silver nanowire. Opt. Exp..

[199] Zhang L., Jin Q., Li G., Zheng J., Yuan Y., Liu A., Yan S., Huang Y. (2019). Plasmonic waveguide on metal nanowires with various symmetry breaking features. Opt. Commun..

[200] Paiva-Marques W.A., Gómez F.R., Oliveira O.N., Mejía-Salazar J.R. (2020). Chiral Plasmonics and Their Potential for Point-of-Care Biosensing Applications. Sensors.

[201] Cheng H., Ge Y., Hu X., Niu X., Wang F., Gao W., Gong Q. (2019). Plasmonic router based on spin-orbital interaction. Opt. Commun..

[202] Ho K.S., Im S.J., Pae J.S., Ri C.S., Han Y.H., Herrmann J. (2018). Switchable plasmonic routers controlled by external magnetic fields by using magneto-plasmonic waveguides. Sci. Rep..

[203] Ando T., Kaji T., Yamaguchi K., Suzuki K., Kamada S., Okamoto T., Mori A., Haraguchi M. (2018). MEMS plasmonic switch with stripe plasmonic waveguide. Jpn. J. Appl. Phys..

[204] Tang C., Gu H., Wang K. (2018). Waffle: A new photonic plasmonic router for optical network on chip. IEICE Trans. Inf. Syst..

[205] Sharma Y., Ghosh R.R., Sapra V., Jalal V., Ahmed K., Dhawan A. Plasmonic switches based on arrays of plasmonic nanostructures surrounded by VO_2_ thin films. Proceedings of the SPIE 10926, Quantum Sensing and Nano Electronics and Photonics XVI.

[206] Salamin Y., Ma P., Baeuerle B., Emboras A., Fedoryshyn Y., Heni W., Cheng B., Josten A., Leuthold J. (2018). 100 GHz Plasmonic Photodetector. ACS Photon..

[207] Gosciniak J., Rasras M. (2019). High-bandwidth and high-responsivity waveguide- integrated plasmonic germanium photodetector. J. Opt. Soc. Am. B.

[208] Huang R., Ji X., Liao Y., Peng J., Wang K., Xu Y., Yan F. (2019). Dual-frequency CMOS terahertz detector with silicon-based plasmonic antenna. Opt. Exp..

[209] Azar N.S., Shrestha V.R., Crozier K.B. Plasmonic Enhancement of Graphene Long-Wave Infrared Photodetectors via Bull’s Eye Concentrator, Optical Cavity and Nanoantennas. Proceedings of the Lasers Electro-Optics Europe European Quantum Electronics Conference (CLEO/Europe-EQEC).

[210] Guo J., Li J., Liu C., Yin Y., Wang W., Ni Z., Dai D. (2020). High-performance silicon-graphene hybrid plasmonic waveguide photodetectors beyond 1.55 *μ*m. Light Sci. Appl..

[211] Kim J.T., Yu Y.-J., Choi H., Choi C.-G. (2014). Graphene-based plasmonic photodetector for photonic integrated circuits. Opt. Exp..

[212] Mousavi S.S., Stöhr A., Berini P. (2014). Plasmonic photodetector with terahertz electrical bandwidth. Appl. Phys. Lett..

[213] Ding Y., Cheng Z., Zhu X., Yvind K., Dong J., Galili M., Hu H., Mortensen N.A., Xiao S., Oxenløwe L.K. (2020). Ultra-compact integrated graphene plasmonic photodetector with bandwidth above 110 GHz. Nanophoton.

[214] Atwater H., Polman A. (2010). Plasmonics for Improved Photovoltaic Devices. Nat. Mater..

[215] Ren M.-X., Wu W., Cai W., Pi B., Zhang X.-Z., Xu J.-J. (2017). Reconfigurable metasurfaces that enable light polarization control by light. Light Sci. Appl..

[216] He Q., Sun S., Zhou L. (2019). Tunable/Reconfigurable Metasurfaces: Physics and Applications. Research.

[217] Rappaport T.S., Xing Y., Kanhere O., Ju S., Madanayake A., Mandal S., Alkhateeb A., Trichopoulos G.C. (2019). Wireless communications and applications above 100 GHz: Opportunities and challenges for 6G and beyond. IEEE Access.

[218] Jiang Z.H., Zhang Y., Xu J., Yu Y., Hong W. (2020). Integrated Broadband Circularly Polarized Multibeam Antennas Using Berry-Phase Transmit-Arrays for Ka-Band Applications. IEEE Trans. Antenn. Prop..

[219] Gangaraj S.A.H., Silveirinha M.G., Hanson G.W. (2017). Berry Phase, Berry Connection, and Chern Number for a Continuum Bianisotropic Material from a Classical Electromagnetics Perspective. IEEE J. Multisc. Multiph. Comp. Technol..

[220] Hannonen A., Partanen H., Tervo J., Setälä T., Friberg A.T. (2019). Pancharatnam-Berry phase in electromagnetic double-pinhole interference. Phys. Rev. A.

[221] Ferreiro-Vila E., García-Martín J.M., Cebollada A., Armelles G., González M.U. (2013). Magnetic Modulation of Surface Plasmon Modes in Magnetoplasmonic Metal-Insulator-Metal Cavities. Opt. Exp..

[222] Belotelov V.I., Kreilkamp L.E., Akimov I.A., Kalish A.N., Bykov D.A., Kasture S., Yallapragada V.J., Gopal A.V., Grishin A.M., Khartsev S.I. (2013). Plasmon-mediated magneto-optical transparency. Nat. Commun..

[223] Mu Q., Fan F., Ji Y., Cheng J., Chang S. (2020). Enhanced terahertz magneto-optical Kerr rotation based on metasurface structure. Opt. Commun..

[224] Hekmatnia B., Naser-Moghadasi M., Khatir M. (2020). Propagation length enhancement in a magneto optic plasmonic Mach-Zehnder isolator using graphene. Opt. Quant. Electr..

[225] Kharratian S., Urey H., Obaşli M.C. (2020). Advanced Materials and Device Architectures for Magnetooptical Spatial Light Modulators. Adv. Opt. Mater..

[226] Girón-Sedas J.A., Gómez F.R., Albella P., Mejía-Salazar J.R., Oliveira O.N. (2017). Giant enhancement of the transverse magneto-optical Kerr effect through the coupling of *ε*-near-zero and surface plasmon polariton mode. Phys. Rev. B.

[227] Moncada-Villa E., Oliveira O.N., Mejía-Salazar J.R. (2019). *ε*-Near-Zero Materials for Highly Miniaturizable Magnetoplasmonic Sensing Devices. J. Phys. Chem. C.

[228] Ghahremanirad E., Olyaee S., Chizari A. Nano-plasmonic thin-film solar cell receiver in visible light communication. Proceedings of the 2016 10th International Symposium on Communication Systems, Networks and Digital Signal Processing (CSNDSP).

[229] Anous N., Abdallah M., Ramadan T., Qaraqe K., Khalil D. (2017). Angle-tolerant hybrid plasmonic filters for visible light communications. Appl. Opt..

[230] Guzatov D.V., Gaponenko S.V., Demir H.V. (2018). Possible Plasmonic Acceleration of LED Modulation for Li-Fi Applications. Plasmonics.

[231] Salamin Y., Baeuerle B., Heni W., Abrecht F.C., Josten A., Fedoryshyn Y., Haffner C., Bonjour R., Watanabe T., Burla M. (2018). Microwave plasmonic mixer in a transparent fibre-wireless link. Nat. Photon..

[232] Real-Time Software-Defined Adaptive MIMO Visible Light Communications (26 July 2017). https://www.intechopen.com/books/visible-light-communications/real-time-software-defined-adaptive-mimo-visible-light-communications.

